# Membrane Assembly during the Infection Cycle of the Giant Mimivirus

**DOI:** 10.1371/journal.ppat.1003367

**Published:** 2013-05-30

**Authors:** Yael Mutsafi, Eyal Shimoni, Amir Shimon, Abraham Minsky

**Affiliations:** 1 Department of Structural Biology, The Weizmann Institute of Science, Rehovot, Israel; 2 Department of Chemical Research Support, The Weizmann Institute of Science, Rehovot, Israel; University of California San Francisco, United States of America

## Abstract

Although extensively studied, the structure, cellular origin and assembly mechanism of internal membranes during viral infection remain unclear. By combining diverse imaging techniques, including the novel Scanning-Transmission Electron Microscopy tomography, we elucidate the structural stages of membrane biogenesis during the assembly of the giant DNA virus Mimivirus. We show that this elaborate multistage process occurs at a well-defined zone localized at the periphery of large viral factories that are generated in the host cytoplasm. Membrane biogenesis is initiated by fusion of multiple vesicles, ∼70 nm in diameter, that apparently derive from the host ER network and enable continuous supply of lipid components to the membrane-assembly zone. The resulting multivesicular bodies subsequently rupture to form large open single-layered membrane sheets from which viral membranes are generated. Membrane generation is accompanied by the assembly of icosahedral viral capsids in a process involving the hypothetical major capsid protein L425 that acts as a scaffolding protein. The assembly model proposed here reveals how multiple Mimivirus progeny can be continuously and efficiently generated and underscores the similarity between the infection cycles of Mimivirus and Vaccinia virus. Moreover, the membrane biogenesis process indicated by our findings provides new insights into the pathways that might mediate assembly of internal viral membranes in general.

## Author Summary

With a particle size comparable to that of small bacteria and a 1.2 Mbp double-strand DNA genome that carries more than 1000 open reading frames, the amoeba-infecting Mimivirus, along with other recently identified members of the *Mimiviridae* family, are the largest and most complex viruses yet identified. The Mimivirus particle includes an internal membrane that underlies an icosahedral capsid. The assembly mechanism of internal membrane during Mimivirus infection remains unclear, as is the case for other viruses containing internal membranes. By using diverse imaging techniques, we showed that membrane biogenesis is an elaborate process that occurs at the periphery of viral factories generated at the host cytoplasm. This multistage process, which includes the formation of open membrane sheets, enables efficient and continuous assembly of multiple Mimivirus progeny. The membrane biogenesis process suggested here provides novel insights into the assembly of internal viral membranes in general.

## Introduction

Nucleocytoplasmic large DNA viruses (NCLDVs), which include *Poxviridae*, *Phycodnaviridae*, *Iridoviridae, Asfarviridae* and *Mimiviridae* [1], replicate and assemble in cytoplasmic inclusions called viral factories. Formation of these elaborate structures that enable spatial and temporal coordination of viral assembly and effective recruitment of host factors, involves massive rearrangement of host cytoskeleton and membranes [2]–[5]. A critical process occurring in these factories is the assembly of inner viral membranes that are present in all NCLDVs. The origin of these membranes, their assembly as well as their number within NCLDV virions remain poorly understood.

Early electron microscopy studies of Vaccinia virus, the prototype of *Poxviridae*, revealed that the immature Vaccinia form (IV) carries a single membrane layer [6], [7]. A single membrane envelope is commonly acquired by viral budding into intracellular compartments or through plasma membranes. Since such processes were not observed during the assembly of IV particles, and as the initial viral membrane structures, dubbed crescents, did not demonstrate a continuity with host membranes, it was proposed that such crescents are generated by a *de novo* synthesis from lipid precursors and have open ends in the cytoplasm [6]. However, the notion of open membrane sheets that expand through *de novo* synthesis is at odds with the common wisdom that membranes are exclusively derived from pre-existing organelles [8], and that open membrane edges are inherently unstable [9]. Subsequent structural studies motivated by this conundrum implied that crescents and IV spheres represent closed structures composed of two tightly apposed bilayers generated through wrapping of collapsed endoplasmic cisternae [8], [10], [11]. The idea that crescents originate from the endoplasmic reticulum (ER) was supported by the finding of an operative pathway from the ER to nascent viral membranes [12], [13]. However, the two-membrane model was challenged by freeze-fracture electron microscopy studies, which demonstrated that IV membrane consists of a single bilayer stabilized by a protein coat [9], [14].

Research on the membrane origin and structure in the African swine fever virus (ASFV) proceeded along a similar course. While initial studies indicated that ASFV virions contain a single membrane layer [15], ensuing observations implied that this NCLDV member contains two internal membranes generated by wrapping of the viral core by collapsed ER cisternae [16]–[18]. Yet, recent investigation revealed that ASFV carries a single lipid bilayer [19]. Notably, another NCLDV member, the phycodnavirus PBCV-1, was also reported to carry a single internal membrane bilayer [20], [21].

If indeed a single membrane bilayer exists, it highlights the uncertainty concerning the pathway by which it is formed [5], [22], [23]. Intrigued by this ambiguity, we set to investigate the mode of membrane generation during the infection cycle of the amoeba-infecting NCLDV Mimivirus [24], [25]. With a 1.2 Mbp dsDNA genome and a particle size of ∼750 nm, the Mimivirus and its relative Megavirus [26], represent the largest viruses documented so far. Mimivirus is composed of a genome encapsulated by a protein core that is surrounded by membrane bilayers [27], which underlie an icosahedral capsid. The capsid is, in turn, covered by closely packed 120 nm-long fibers that form a dense matrix [27]–[29]. The large size of the Mimivirus membrane layers, along with the fact that almost 1000 virus progeny are generated within each infected cell during a relatively short (12–14 hours) infection cycle [24], highlight questions concerning the source of lipid components required for the viral membranes as well as the mechanisms responsible for a precise and rapid assembly of these membranes.

To investigate membrane assembly during Mimivirus infection, we used fluorescence microscopy, immunolabeling, freeze-fracture cryo-Scanning Electron Microscopy and Scanning-Transmission Electron Microscopy (STEM) tomography of cryo-preserved samples. STEM tomography is a novel technique that provides higher contrast than conventional electron tomography and enables data acquisition from thicker (>250 nm) samples [30]. Our studies reveal that at late stages of Mimivirus infection, ER-like cisternae are recruited to the periphery of the viral factory. Numerous vesicles that appear to bud out from these cisternae fuse into multivesicular structures that eventually rupture to generate open, single-membrane sheets reminiscent of those detected during Vaccinia assembly [31]. An angular vertex is subsequently assembled, leading to the formation of icosahedral capsids. These observations elucidate the process by which generation of multiple Mimivirus progeny can proceed continuously and efficiently. Moreover, our findings imply that this pathway may represent a common mechanism for the assembly of inner viral membranes as well as for the formation of membrane structures during fundamental cellular processes.

## Results

### Structure of the Mimivirus Virion and Its Intracellular Factory

**Figure 1:**
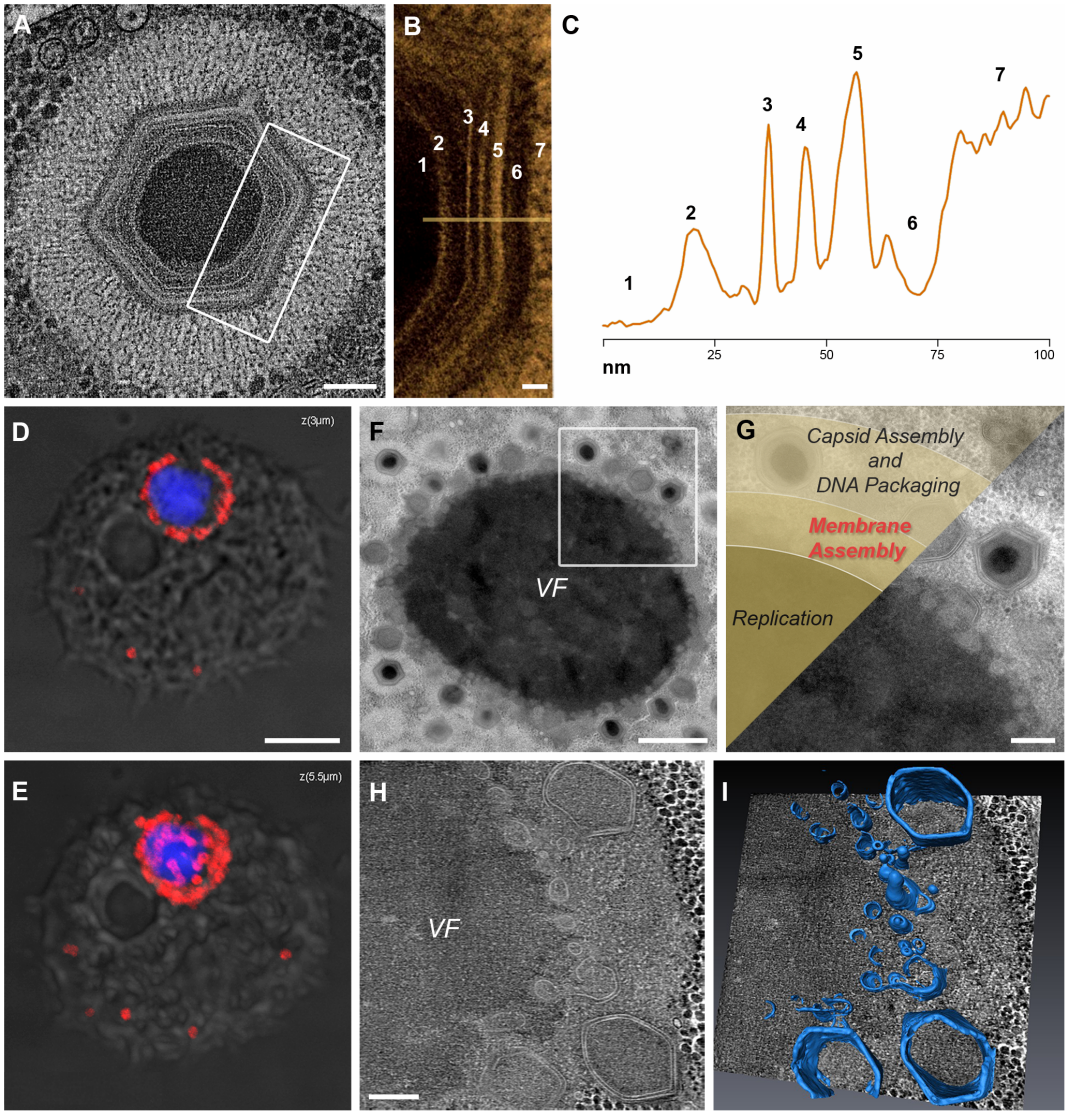
Mimivirus membrane organization and membrane assembly. **A**. 0.8 nm slice from STEM tomography of a mature intracellular Mimivirus particle. **B**. Magnified representation of the region delineated in (A). The layers comprising the Mimivirus virion are: 1: DNA core; 2: Core wall; 3: Inner membrane; 4: A layer previously proposed to represent an outer membrane [27]; 5: Inner capsid shell; 6: Outer capsid shell; 7: Fibers (Movie S1). **C**. Density plot of the various layers along the line drawn in (B). Digits correspond to those depicted in B. **D**, **E**. Confocal images of a 0.5 µm z-section (D) and a projection composed of 11 0.5 µm z-sections (E) showing the Mimivirus factory in the cytoplasm of an 8 hours PI cell revealing viral DNA (DAPI, blue) surrounded by assembling viruses (anti-fibril antibody, red). **F**. TEM of a factory (VF) in an 8-hour PI cell showing icosahedral capsids at the factory periphery. **G**. Magnification of the delineated region in (F), depicting viral assembly zones overlying the TEM image. Three distinct zones are proposed: viral replication, membrane assembly, and capsid assembly zones. **H**, **I**. 7.5 nm digital slice derived from STEM tomography of 280 nm-thick section (H) and 3-dimensional surface rendering (I) reveal that the membrane assembly region consists of an elaborate membrane network (Movie S2). Scale bars: 200 nm in A, H; 20 nm in B; 5 µm in D, E; 2 µm in F and 500 nm in G.

Since our studies of membrane biogenesis during Mimivirus infection were carried out using mainly STEM tomography, we analyzed the structure of mature intracellular Mimivirus virions using the same technique in order to enable direct comparison (Fig. 1A–C; Movie S1). Our observations corroborate results derived from single-particle analyses [27] by revealing the presence of two layers that surround the core wall (layers 3 and 4 in Fig. 1B, C). However, our results are inconsistent with the notion that the outer layer (layer 4) represents a membrane, as discussed below.

We have previously shown that shortly following Mimivirus infection, replication of viral genomes that are released into the host cytoplasm, is initiated, leading to the formation of replication centers whose number per host cell correlates with the multiplicity of infection [32]. At ∼6 hours post infection (PI), replication centers coalesce into a single viral factory in which Mimivirus progeny are generated. Fluorescence microscopy and TEM indicate a burst-like capsid formation at the periphery of the factory at 8 hours PI (Fig. 1D–F). Previous studies [33] implied that the factory core is surrounded by a membrane assembly site (Fig. 1G). This conjecture is corroborated by our STEM tomography studies, which demonstrate that this region consists of a highly elaborate membrane network, including multiple vesicles and membrane sheets (Fig. 1H, I; Movie S2).

Our TEM sample preparation involves high-pressure-freezing followed by freeze substitution with acetone (see Materials and Methods), a technique shown to be the method of choice for the preservation and resolution of membrane structures in studies of ASFV and Vaccinia infection cycles [19], [31]. However, since this method involves exposure to organic solvents, we sought to further ascertain that membrane structures are accurately preserved. We thus used cryo-scanning electron microscopy to inspect membrane assembly zones in specimens prepared by freeze-fracture, a method that preserves samples in their native state as no organic solvents or chemical fixatives are used (see Materials and Methods). Membrane structures detected by this technique, including sheets associated with assembling capsids and vesicles, were similar to those observed in samples prepared by high-pressure-freezing followed by freeze substitution (Fig. S1), thus validating our TEM studies.

### Host ER Membranes Accumulate at Close Vicinity to Viral Factories

**Figure 2:**
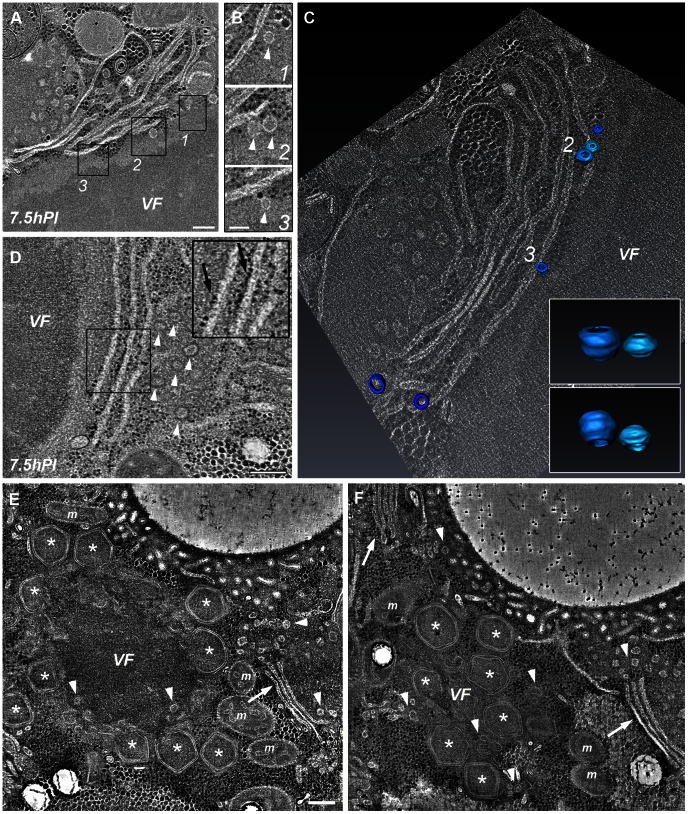
Cisternae and vesicles apposing viral factories at the onset of capsid assembly. **A**. 10 nm digital slice derived from a 280 nm-thick STEM tomogram of a 7.5-hour PI viral factory. The slice reveal cisternae (arrows) and clusters of ∼70 nm vesicles (arrowheads) at close vicinity to the viral factory (VF). **B**. Magnification of the delineated regions in (A). **C**. A different digital slice from the STEM tomogram shown in (A), with 3-dimensional surface rendering of vesicles; numbers correspond to the regions delineated in (A) and (B). The insets depict magnified 3-dimensional surface rendering of the vesicles from region 2 at two different angles, demonstrating the spherical structure of the vesicles (Movie S3). **D**. 10 nm digital slice from a different 280 nm-thick STEM tomogram of a 7.5-hour PI viral factory, showing membrane structures surrounded by multiple vesicles near viral factories (Movie S4). The inset demonstrates that membrane structures apposing the factories are studded with ribosomes (black arrows). **E**, **F**. Two STEM tomogram slices of an 8-hour PI viral factory that are derived from serial sections and are located 480 nm apart. These tomograms reveal that as Mimivirus progeny are assembled, host cisternae are excluded from the membrane assembly zone (Movies S5, S6). Scale bars: 200 nm in A, D; 100 nm in B; 250 nm in E.

As indicated above, fluorescence and TEM studies reveal the presence of fully assembled icosahedral capsids at the periphery of viral factories at ∼8 hours PI. To obtain insights into earlier stages of capsid generation, STEM tomography analyses were conducted on thick (280–320 nm) sections of infected cells at 7.5 hours PI. These analyses revealed membrane cisternae localized at close proximity to the factories (Fig. 2A–D; Movies S3, S4). Notably, the cisternae do not enwrap the whole factory but rather are detected at discrete sites that consistently coincide with regions where angular structures are detected. This observation, along with the finding that neither cisternae nor angular structures are present in earlier PI times, imply a causal correlation between membrane cisternae and capsid assembly. In addition, cisternae are regularly associated with multiple uniformly sized (∼70 nm) vesicles that appear to be budding from the cisternae.

Whereas at 7.5 hours PI cisternae are detected at 50 to 100 nm from the edge of the factory core, at 8 hours PI, when partially and fully assembled icosahedral capsids already surround the entire viral factory, these membrane structures are observed at significantly larger distances (∼500 nm) from the factory core, apparently excluded by newly assembling capsids (Fig. 2E, F; Movies S5, S6). However, multiple ∼70 nm vesicles are present near the cisternae as well as within the inner, membrane assembly zone, thus supporting the notion that the vesicles are derived from the cisternae. Notably, this finding implies that these vesicles, which are capable of reaching the membrane assembly zone due to their small size, act as a vehicle that enables continuous supply of lipid components required for virion assembly.

**Figure 3:**
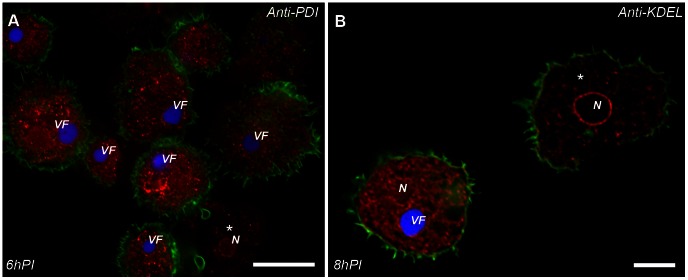
Mimivirus infection induces synthesis of host ER. *A. polyphaga* cells were infected at 10 MOI (multiplicity of infection), fixed at the indicated PI time points, and stained with phalloidin-488 actin probe (green) to trace cell border and DAPI (blue). **A**. Cells stained with mouse anti-protein disulfide isomerase (red), a soluble protein residing in the ER lumen. **B**. Cells stained with KDEL retention peptide that specifically labels RER membrane proteins. In both staining experiments, ‘*’ indicate non-infected cells, revealing that Mimivirus infection results in a massive redistribution of RER markers. N: nucleous; VF: viral factory. Due to the large amount of dsDNA within VFs relative to that present in cell nuclei, the nuclei are not visible at DAPI exposure levels required to detect VFs. Scale bars: 15 µm in A; 5 µm in B.

While the presence of membrane layers has been unequivocally demonstrated in all NCLDV, their source remains controversial. Our TEM studies demonstrate that the cisternae apposed to viral factories are occasionally studded with ribosomes (Fig. 2D; Movie S4), thus revealing a characteristic appearance of rough endoplasmic reticulum (RER). The origin of the Mimivirus membranes was also investigated by using antibodies against common endoplasmic reticulum markers. These included protein disulfide isomerase (PDI), a soluble protein residing in the ER lumen, and the KDEL retention peptide specifically labeling RER proteins. Substantial redistribution of these markers in infected host-cells is observed at 6 hours PI (Fig. 3A, B). These observations, along with the RER-like appearance of the cisternae apposing the viral factories, infer that cisternae are derived from the host ER network, yet this conjecture needs to be further investigated.

### Multivesicular Bodies and Open Sheets Are Generated at the Membrane Assembly Zone

**Figure 4:**
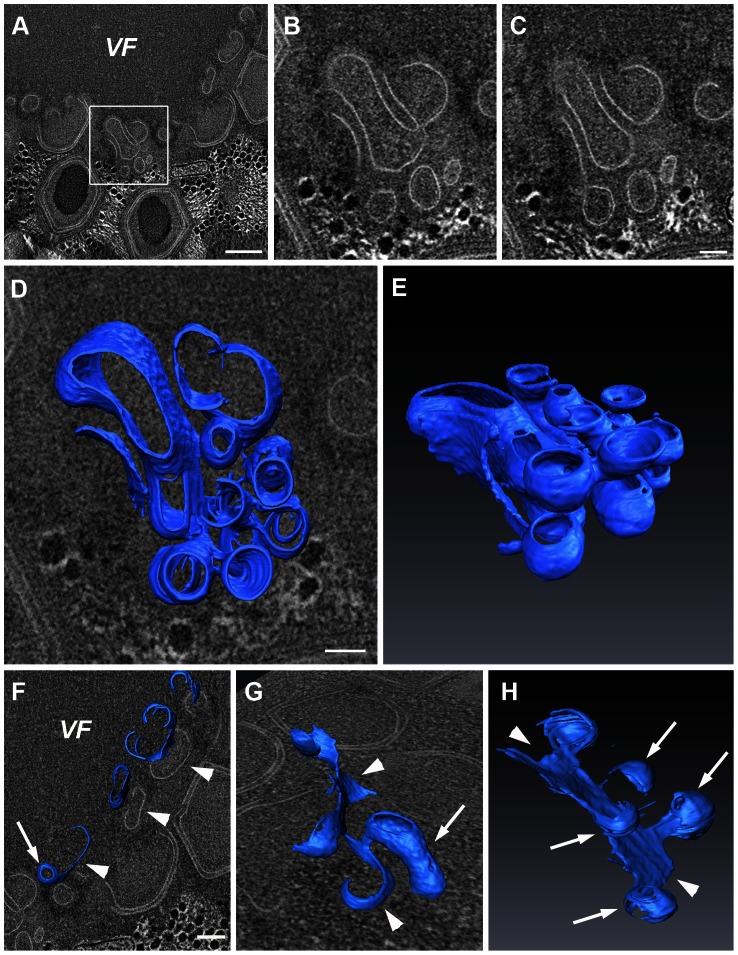
Multivesicular bodies and open sheets in the membrane assembly zone. **A**. 10 nm digital slice derived from a 280 nm STEM tomogram of an 8-hour PI viral factory (VF). **B**, **C**. Two magnified 10 nm slices of the region delineated in A that are 20 nm apart in the z-axis. **D**, **E**. Top and side views, respectively, of a 3-dimensional surface rendering representation derived from the tomogram shown in A–C, highlighting the multi-vesicle nature of the body and the vesicular connectivity within these bodies. **F**–**H**. 3-D surface rendering from a STEM tomogram of a different 8-hour PI VF, revealing a cluster of open membrane sheets (arrowheads in F) connected to membrane tubules (arrowheads and arrow in g), and to 70 nm vesicles (arrowheads and arrows in H). See Movies S7, S8. Scale bars: 200 nm in A; 100 nm in B–H.

At 8 hours PI, massive generation of Mimivirus capsids and maturation of virion progeny occur concomitantly in the inner, membrane biogenesis zone, and at the outer capsid assembly and DNA packaging zone. STEM tomography reveals the presence of several substructures within the membrane biogenesis zone at this stage. In addition to multiple vesicles and angularly-shaped assemblies described above, two prominent substructures are regularly detected: multivesicular bodies and open membrane sheets. Multivesicular bodies comprise of multiple vesicles that reveal similar size and curvature characterizing free vesicles detected in this region as well as near the cisternae, in addition to larger tubular structures (Fig. 4A–E; Movies S7, S8). However, these vesicles are interconnected and share their lumen. In addition, large open membrane sheets that are connected to vesicles and to tubular structures (Fig. 4F–H; Movie S9) are regularly detected.

**Figure 5:**
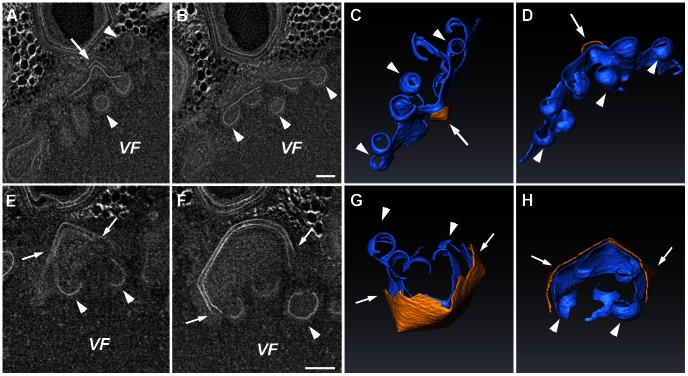
Assembly of icosahedral capsids on top of membrane sheets. **A**, **B**. 10 nm digital slices 40 nm apart derived from 320 nm STEM tomogram of 8-hour PI VFs, revealing formation of angular structures by a layer lying on top of open membrane sheets (arrow) that are surrounded by vesicles (arrowheads). **C**, **D**. Two angles of 3-dimensional surface rendering presentations derived from panels A and B, showing an early assembling capsid. Membrane sheets and vesicles (arrowheads) are depicted in blue and newly generated angular structures are indicated in yellow (arrows). **E**, **F**. Two 10 nm digital slices from a later assembly stage, revealing the progression of angular structures (arrows) into truncated icosahedral morphologies and open membrane sheets still connected to vesicles (arrowheads). **G**, **H**. Two different angles of 3-dimensional surface rendering presentations of an assembling icosahedral capsid (yellow; arrows) on top of a membrane layer (blue; arrowheads). The corresponding movies are S10 and S11 for A–D, and Movie S12 for E–H. Scale bars: 100 nm.

Formation of multivesicular bodies and open membrane sheets is closely followed by the generation of angular structures (Fig. 5A–D; Movies S10, S11) that represent the precursors of icosahedral capsids. The emergence of angular morphologies is accompanied by the formation of a second layer on top of the membrane open sheets, that is, on the side pointing away from the factory core. This angular outer layer progressively evolves into icosahedral morphology, shaping the underlying membrane layer into the same geometry (Fig. 5E–H; Movie S12).

### Mimivirus Capsid Protein L425: A Potential Scaffolding Protein

We sought for a protein that might act as scaffold during Mimivirus membrane and capsid assembly. The Vaccinia D13 protein plays a critical role in Vaccinia assembly by generating a honeycomb scaffold on the convex side of open membrane sheets [9], [14], [22], [31], [34]–[36]. On the basis of a partial sequence similarity to D13L, we speculated that the Mimivirus hypothetical major capsid protein encoded by the L425 open reading frame [24] might act as such a scaffold, a conjecture supported by the recent modeling of L425 [34].

**Figure 6:**
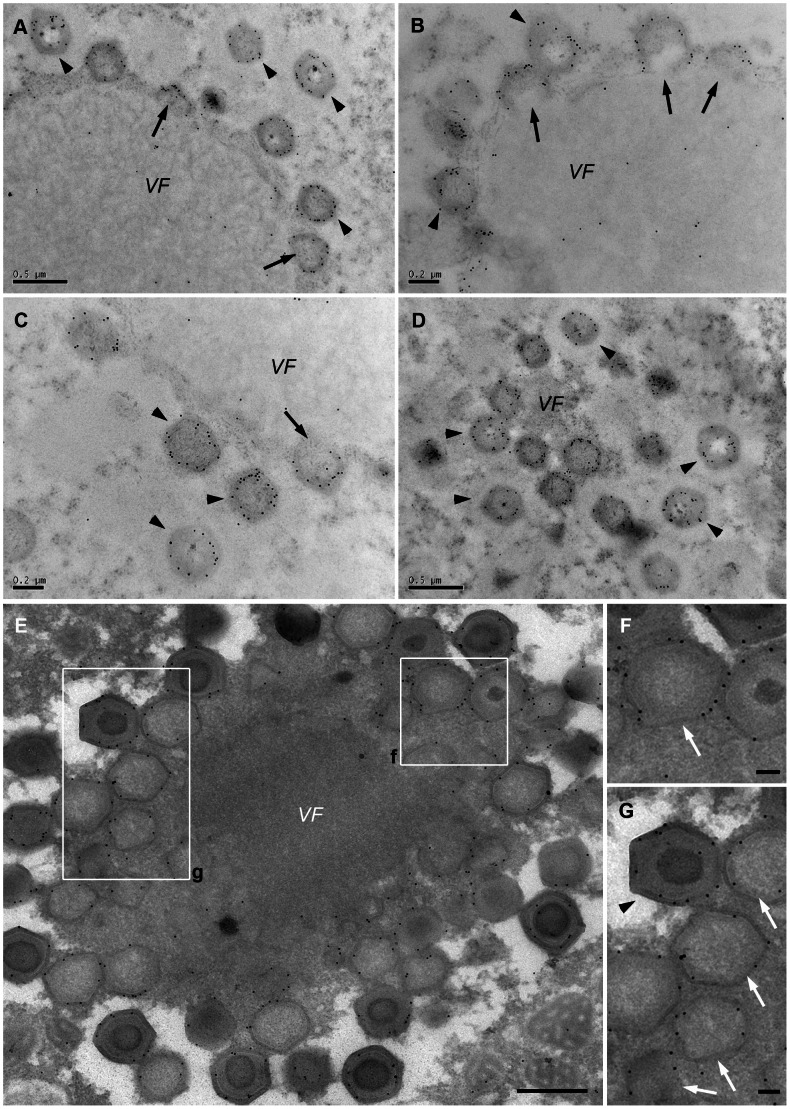
Mimivirus L425 protein acts as an icosahedral capsid scaffolding protein. **A**–**D**. 8-hours PI cells were cryo-preserved and exposed to rabbit anti-L425 antibodies, followed by exposure to gold-conjugated goat anti-rabbit antibody, revealing that pre-assembled capsids (arrows) as well as fully assembled icosahedral capsids (arrowheads) include L425. **E**–**G**. Infected cells processed by the immuno-TEM Tokuyasu method. As is the case for cryo-preserved specimens, anti-L425 antibodies specifically label the external layer of the open sheet membrane. Scale bars: 500 nm in A, D and E; 200 nm in B–C; 100 nm in F–G.

To evaluate this idea we raised antibodies against L425 in both mice and rabbits by using a mixture of three peptides derived from L425 that were estimated as particularly immunogenic. The resulting antibodies recognized a single ∼70 kDa band in lysates of both infected host cells and purified viruses (Fig. S2), thus confirming that these antibodies interact with L425, whose calculated weight is 67.27 kDa. Immunolabeling with anti-L425 antibodies of both cryo-preserved (Fig. 6A–D) and chemically-fixed (Fig. 6E–G) sections of 8 hours PI cells, but not of any cell sections derived from earlier PI time points, resulted in clear labeling of angular structures, of assembling icosahedral morphologies as well as of fully assembled capsids (Fig. 6 and Fig. S2).

### Assembling Capsids Undergo Membrane Trimming

**Figure 7:**
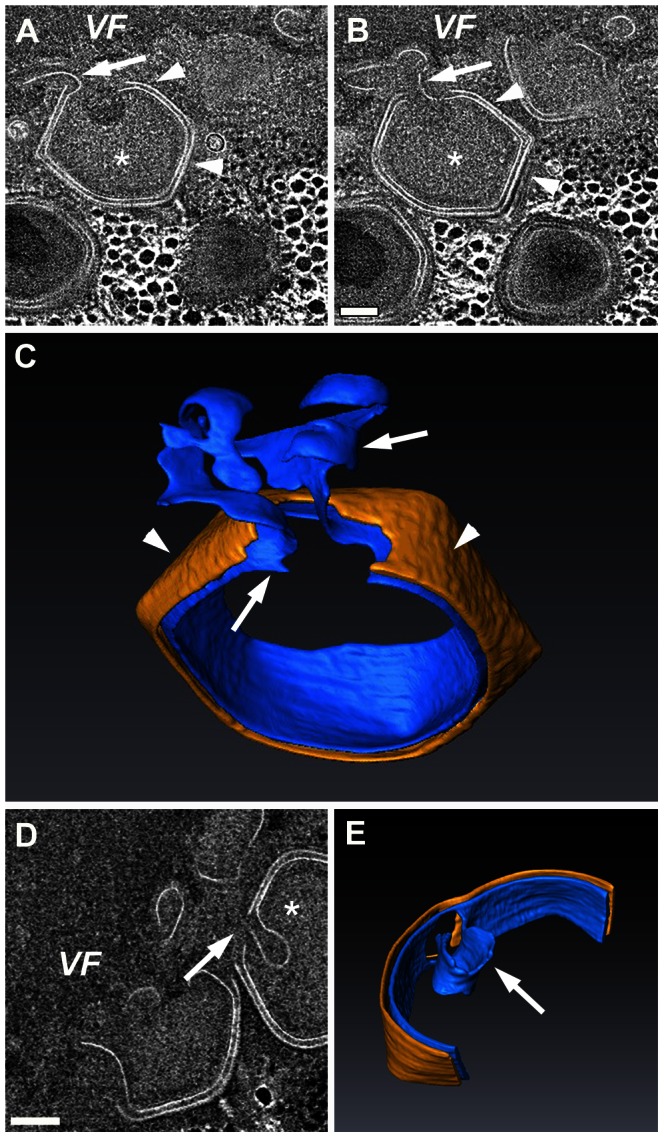
Trimming of open-sheet membrane surplus. **A**, **B**. Two 10 nm digital slices derived from a 320 nm-thick STEM tomogram from the periphery of an 8-hour PI Mimivirus VF, revealing that the inner viral membrane underlying an icosahedral capsid layer (arrowheads) is connected to external membrane sheets (arrows). **C**. 3-D surface rendering view of icosahedral capsids (arrowheads; yellow) and inner membrane (blue) that are still connected to external open membrane sheets (arrows; blue). **D**, **E**. 10 nm digital slice (D) and a 3-dimensional surface rendering view (E) of fully assembled Mimivirus virions in which excess external membrane is trimmed (arrow in E), leaving a ∼20 nm DNA entry portal. See Movies S13, S14. Scale bars: 100 nm.

Our findings imply that the inner Mimivirus membrane is derived from large open membrane sheets that are continuously generated through vesicle fusion and rupture. This process raises the question how is the final size of this inner membrane precisely constrained and determined? Our high-resolution 3-dimensional STEM tomography studies reveal that throughout the process of capsid formation, long membrane ‘overhangs’ consisting of open sheets are connected to the inner membrane layer (Fig. 7A–C; Movies S13, S14). Such surplus sheets appear to be trimmed at a late stage of capsid assembly, during which a 20 nm portal that enables subsequent genome entry and packaging [28] is generated (Fig. 7D, E).

## Discussion

In large DNA viruses the origin of membrane layers, their numbers, as well as the mechanisms responsible for their assembly remain controversial [5], [23], [37]–[39]. The current leading view is that these viruses carry a single inner membrane bilayer [9], [19], [21], [22], raising the question how is such a single bilayer generated. Specifically, whereas formation of two membranes can be straightforwardly assigned to wrapping of collapsed ER cisternae, a single bilayer requires either single-layer membrane precursors or the loss of one ER membrane layer by yet unidentified mechanisms. Insights into these questions were recently provided by cryo-EM and electron tomography of Vaccinia-infected cells [31], which revealed that Vaccinia crescent precursors are single membrane sheets generated through rupture of the host ER membrane network.

**Figure 8:**
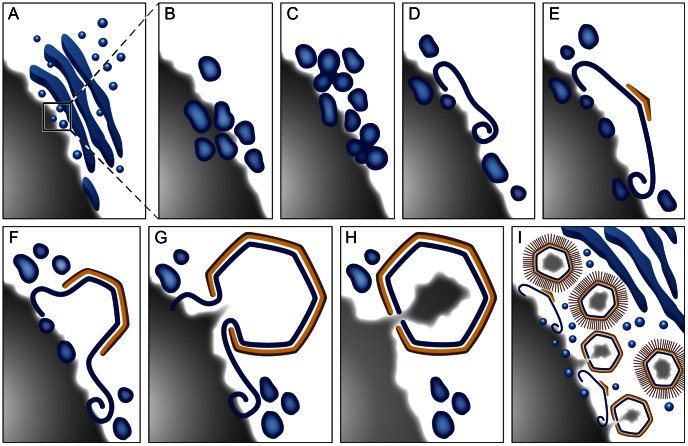
Model of Mimivirus membrane and capsid assembly. **A**. Host cisternae, from which ∼70 nm vesicles bud out, are recruited to early (7.5-hour PI) viral factories. **B**. Zoom-in of multiple ∼70 nm vesicles reaching membrane assembly zone. **C**. Vesicles fuse into multivesicular bodies. **D**. Multivesicular bodies generated at 8-hour PI rupture to form large open membrane sheets that act as precursors for the inner Mimivirus membrane. The open sheet is expanding by fusing with additional incoming vesicles. **E**. An icosahedral vertex is generated on top of the open membrane sheets through the assembly of structural Mimivirus capsid proteins in a process involving the L425 protein. **F**. Upon assembly of the capsid, the inner membrane layer is shaped into icosahedral morphology. **G, H**. Membrane overhangs consisting of the open membrane sheets are suggested to prevent premature closure of icosahedral capsids, thus enabling formation of a DNA-encapsidating portal [28]. **I**. As multiple Mimivirus capsid progeny are generated, large host cisternae that act as viral lipid reservoir are excluded from the membrane assembly zone, thus highlighting the need for small, ∼70 nm vesicles capable of reaching this zone, to enable continuous viral assembly. The model underscores the notion that Mimivirus factories represent ‘production lines’ where all stages of viral generation occur *simultaneously*. Gray: viral DNA; Blue: membrane network; Yellow: capsids.

On the basis of the observations reported here we suggest a structural model for the multiple stages of membrane biogenesis during Mimivirus infection cycle (Fig. 8). Generation of Mimivirus inner membrane is initiated at ∼7.5 hours PI by the recruitment of host cell membrane cisternae to Mimivirus factories (Fig. 8A), which are formed in the host cytoplasm [25], [28], [32]. The idea that cisternae are actively recruited to the factory needs, however, to be further assessed as a passive pathway consisting of encounters of expanding cytoplasmic factories with host cisternae is plausible. Abundant uniformly sized (∼70 nm) vesicles are regularly detected at close proximity to the cisternae (Fig. 8A, B). We suggest that these vesicles are continuously budding from cellular cisternae that act as a reservoir for viral membrane components.

As vesicles accumulate in the membrane assembly zone, they fuse into large multivesicular bodies, within which vesicles are interconnected and share a common lumen (Fig. 8C). The idea that multivesicular bodies are generated by fusion of vesicles derived from host membrane cisternae is based on the observation that these bodies are closely surrounded by multiple vesicles. This notion is further supported by the finding that the size and curvature of the vesicular structures composing multivesicular bodies are identical to those characterizing free single vesicles. In addition to fused vesicular structures, a prominent feature revealed by multivesicular bodies is large open membrane sheets consisting of a single membrane bilayer (Fig. 8D). These open sheets, which appear to form through rupture of the multivesicular bodies, continuously expand as additional vesicles fuse to the multivesicular complexes and subsequently rupture, as implied by the evident continuity between open sheets and the ∼70 nm vesicles. As is the case for membrane biogenesis in Vaccinia [31], the mechanism that promotes rupture and the factors that stabilize the resulting open membrane sheets remain to be identified.

Assembly of icosahedral capsids is initiated by the formation of a layer on top of the membrane open sheets that is accompanied by the emergence of prominent angular structures consistently pointing away from the viral factory (Fig. 8E). These angular structures expand to form complete icosahedral capsids, shaping the underlying membrane layer into the same morphology (Fig. 8F, G). Immunolabeling analyses indicate that the angular structures as well as mature icosahedral capsids include the hypothetical major Mimivirus capsid protein L425. This protein reveals significant similarity to the Vaccinia D13 [34], which acts as scaffold protein [9], [14]. Yet, whereas D13 protein disassembles upon Vaccinia maturation [35], our immunolabeling analyses reveal that L425 remains a constituent of mature Mimivirus virions, in agreement with previous reports [40].

Throughout the process of capsid formation, long membrane ‘overhangs’, consisting of open sheets that are connected to the evolving inner membrane layer, are present (Fig. 8G). We suggest that, by preventing premature closure of the icosahedral capsids, these overhangs, which are eventually trimmed, play a role in the generation and stabilization of a 20 nm portal through which packaging of the Mimivirus genome proceeds (Fig. 8H). Significantly, in light of the notion that the first icosahedral vertex to be assembled is the DNA-release portal (the stargate) [28], the model proposed here (Fig. 8-H) provides a direct interpretation to the observation that the DNA-packaging portal is generated at the opposite side to the stargate. Moreover, our membrane biogenesis and capsid assembly model highlights the dynamic nature of these processes that is essential for ongoing generation of multiple progeny virions. Specifically, as capsid assembly proceeds and new capsid layers are generated at the periphery of the viral factory, large host cisternae are excluded from the inner zone where membrane biogenesis takes place. In contrast, small vesicles are still capable of reaching the membrane assembly zone, thus allowing for continuous supply of lipid components (Fig. 8I). Subsequent vesicle fusion into large multivesicular bodies at the inner membrane biogenesis zone and their eventual rupture enable formation of the large inner viral envelope (>400 nm in diameter) from the small ∼70 nm vesicles. Notably, the results reported here are inconsistent with the notion that Mimivirus virions include two internal membrane layers [27]. Rather, our observations imply that this layer represents a scaffolding proteinaceous shell and that the Mimivirus virion contains a single membrane layer, as apparently is the case for other NCLDV members such as Vaccinia, ASFV and PBCV-1 viruses (see Introduction for more details).

The proposed model should be viewed as an initial framework for highlighting underlying questions and defining future research directions. First and foremost is the origin of inner membrane layers that are present in all NCLDV virions. For Vaccinia membrane biogenesis, ER-Golgi intermediate compartment (ERGIC) membranes were suggested as a source of viral membranes [10], [11], [41], but other studies implied that Vaccinia inner membrane derives from host ER [12], [13], [22], [39]. ER network was also proposed as the source of the ASFV internal membrane [16]–[18], [42]. Our findings, demonstrating that membrane cisternae accumulating near the factories are studded with ribosomes and that Mimivirus infection is accompanied by massive redistribution of ER markers, infer that Mimivirus membranes derive from host ER, but this conjecture remains to be further evaluated. A fundamental issue that needs to be elucidated concerns the proteins that, in addition to L425, are involved in the assembly stages described here.

Our observations reveal how multiple Mimivirus progeny can be continuously assembled at the periphery of viral factories. The assembly model depicted in Figure 8 underscores the notion that these factories can be considered as efficient ‘production lines’ where, at any given moment all stages of viral generation, including membrane biogenesis, capsid assembly and genome encapsidation, are occurring *concomitantly*. Moreover, previous reports underscored the similarity between the replication cycles of Vaccinia and Mimivirus by demonstrating that Mimivirus infection takes place entirely in the host cytoplasm [28], [32], as is the case for Vaccinia [43], [44]. Our current findings extend the notion of a physiological similarity between Vaccinia and Mimivirus by demonstrating that membrane biogenesis in both viruses proceeds through the formation of open membrane sheets through rupture of vesicular structures. The question whether this pathway is shared by other viruses as well as by cellular processes that involve membrane assembly represents another fascinating research direction.

## Materials and Methods

### Cells and Viruses

*Acanthamoeba polyphaga* (AP) and Mimivirus were obtained from Prof. D. Raoult (U. Méditerranée, Marseille, France). AP Cells were grown in PYG medium.

### Sample Preparation for TEM Studies

Infected AP cells at various post infection (PI) times were spun at 1,000 rpm for 5 minutes and 4–6 µl of condensed pellets were cryo-immobilized as reported [28]. Thin (60–80 nm) sections were mounted onto 100–200 mesh copper grids and post stained with 2% uranyl acetate and Reinold's lead citrate. Samples were imaged in a FEI Tecnai T-12 TEM operated at a 120 kV. Images were recorded with an Eagle 2K×2K FEI CCD camera (Eindhoven, the Netherlands).

### STEM Tomography

Thick sections (250–400 nm) of embedded samples were transferred to 150-mesh copper grids decorated with 12 nm gold markers on both sides and coated with thin carbon film (Edwards). Tomograms were acquired with FEI Tecnai TF20 TEM operated at 200 kV. Automatic sample tilting, focusing and image shift correction were performed with Xplore3D software (FEI). Tomograms were acquired from −60° to +60° double-tilt series with 1° increments, with Gatan bright-field detector in the nanoprobe mode. 3D reconstructions were computed from tilt series using a weighted back-projection IMOD package. Tomograms were post processed either with a medium or a smoothing filter. Volume segmentation, visualization, and movies creation were conducted with Avizo 6.3 image processing package (Visualization Science Group, Burlington, MA, USA). Segmentations were performed by automated as well as guided segmentation modes.

### Immunofluorescence

Indirect immunofluorescence studies were performed as previously described [32]. Briefly, cells were seeded on glass coverslips, infected with Mimivirus particles at MOI of 10 and incubated for the indicated times before fixing and treating with various antibodies. Following incubation with Cy3-conjugated donkey anti-mouse IgG (Jackson Immunoresearch), samples were counterstained with DAPI. Fluorescence images were obtained with a Deltavision system (Applied Precision). Images were de-convoluted with the conservative SoftWorx package using high noise filtering.

### Immunoelectron Microscopy

Lowicryl HM-20-embedded samples were freeze-substituted, slowly warmed to −30°C, infiltrated with increasing concentrations of HM-20 and polymerized at −30°C with UV light. Infected AP cells were sectioned (100–120 nm thick) on formvar-coated nickel 200 mesh grids. Following treatment with blocking solution grids were incubated with a rabbit anti-L425 antibody. Grids were rinsed with PBS and incubated with 10-nm-gold conjugated goat anti-rabbit. Samples were visualized using an FEI Spirit Tecnai T-12. For the Tokuyasu immunolabeling method, samples were prepared as previously reported [32]. Mouse and Rabbit anti-L425 antibodies were obtained by immunization with a mixture of three synthetic peptide sequences derived from the L425 sequence and chosen on the basis of their high immunogenic patterns.

## Supporting Information

Figure S1 **Membrane sheets and vesicles are associated with assembling capsids at 8hPI at the Viral Factory periphery.** *A. polyphaga* cells were infected at MOI of 10 and fixed by high pressure freezing. Cells were further processed for TEM studies (A,C) or for freeze fracture cryo-SEM studies (B, D). **A–D**. Membrane components are found at the interface between the VF and the assembling icosahedral capsids (white asterisks) in the forms of open continuous sheets (A, B) as well as vesicles (C, D). Scale bars are 100 nm.
(TIF)

Figure S2 **Characterization of the anti-L425 antibody by Western blot.** **A**. *A. polyphaga* cells were mock infected (lane 1- Con), or infected in an MOI of 10 (lanes 3, 4). Cells were scraped from the dishes at 8hPI (lane 3) and 10hPI (lane 4) and resuspended in cold lysis buffer. Total protein concentration was measured and identical protein amounts (20 µgr per lane) were loaded on 10% SDS-PAGE. The samples were blotted onto nitrocellulose membrane. L425 protein was detected by Rabbit anti-L425, followed by HRP-conjugated antibodies and ECL reaction. Left: molecular size markers in kDa. **B, C**. Immuno-TEM studies of chemically fixed cryo-thawed sections of 8hPI assembling viral factory labeled with rabbit anti-L425 antibodies. Empty, fully assembled capsids (arrowheads in B) and encapsidating particles (arrow in B) are labeled with the anti L425. The L425 is also found in fully mature virions (C). Scale bars: 100 nm.
(TIF)

Movie S1 **Tomogram of a mature Mimivirus particle.** Dual axis STEM tomogram of a 320 nm thick section. The Movie corresponds to panels 1A, B. The movie was created with the slicer mode in the IMOD program, where each section is a 5.2 nm averaged view of 10 subsequent orthographic sections.
(AVI)

Movie S2 **Tomogram and 3D surface rendering of the membrane assembly zone of an 8hPI Viral Factory.** Dual axis STEM tomogram of a 280 nm thick section. The movie corresponds to panels 1H, I. The movie was created with the Aviso movie-maker platform. The membrane components in the membrane assembly zone were labeled in blue.
(MPG)

Movie S3 **Tomogram of membrane cisternae detected at the periphery of a 7.5hPI Viral Factory.** Dual axis STEM tomogram of a 280 nm thick section. The Movie corresponds to panels 2A–C. The movie was created with the slicer mode in the IMOD program, where each section is a 10.27 nm averaged view of 10 subsequent orthographic sections.
(AVI)

Movie S4 **Tomogram of membrane cisternae detected at the periphery of a 7.5hPI Viral Factory.** Dual axis STEM tomogram of a 280 nm thick section. The Movie corresponds to panels 2D. The movie was created with the slicer mode in the IMOD program, where each section is a 10.27 nm averaged view of 10 subsequent orthographic sections.
(AVI)

Movie S5 **Tomogram of an assembling 8hPI Viral Factory and its neighboring membrane cisternae.** Dual axis STEM tomogram of a 160 nm thick section from a serial 5 section tomogram. The Movie corresponds to panels 2E. The movie was created with the slicer mode in the IMOD program, where each section is a 7 nm averaged view of 5 subsequent orthographic sections.
(AVI)

Movie S6 **Tomogram of an assembling 8hPI Viral Factory and its neighboring membrane cisternae.** Dual axis STEM tomogram of a 160 nm thick section from a serial 5 section tomogram. The Movie corresponds to panels 2F. The movie was created with the slicer mode in the IMOD program, where each section is a 7 nm averaged view of 5 subsequent orthographic sections.
(AVI)

Movie S7 **Tomogram of the membrane zone of an assembling 8hPI Viral Factory at high magnification.** Dual axis STEM tomogram of a 280 nm thick section. The Movie corresponds to panels 4A–F. The movie was created with the slicer mode in the IMOD program, where each section is a 3.64 nm averaged view of 5 subsequent orthographic sections.
(AVI)

Movie S8 **Tomogram and 3D surface rendering of the Multivescular bodies found at the membrane assembly zone.** The movie corresponds to panels 4D, E. The movie was created with the Aviso movie-maker platform. The membrane components of the multivesicular body were labeled in blue, demonstrating its complex structure.
(MPG)

Movie S9 **Tomogram of the membrane zone of an assembling 8hPI Viral Factory at high magnification.** Dual axis STEM tomogram of a 280 nm thick section. The 3D surface rendered membrane sheet shown in panel 4G was generated on the basis of the tomogram. The movie was created with the slicer mode in the IMOD program, where each section is a 3.64 nm averaged view of 5 subsequent orthographic sections.
(AVI)

Movie S10 **Tomogram of the membrane zone of an 8hPI Viral Factory at high magnification.** Dual axis STEM tomogram of a 320 nm thick section. The 3D surface rendered membrane sheet shown in panels 5A–D was generated on the basis of the tomogram. Movie S10 was created with the slicer mode in the IMOD program, where each section is a 3.64 nm averaged view of 5 subsequent orthographic sections.
(AVI)

Movie S11 **3D surface rendering of an initial assembling capsid.** Dual axis STEM tomogram of a 320 nm thick section. The 3D surface rendered membrane sheet shown in panels 5A–D was generated on the basis of the tomogram. Movie S11, demonstrating the initial steps of the creation of the capsid (yellow) on top of the open membrane sheet (blue). The movie was created with the Aviso movie-maker platform.
(MPG)

Movie S12 **Tomogram and 3D surface rendering of an assembling capsid.** The movie corresponds to panels 5E–H. The movie was created with the Aviso movie-maker platform. The capsid (yellow) layer is generated on top of the open membrane sheet (blue) shaping the membrane into an icosahedral structure.
(MPG)

Movie S13 **Tomogram of the membrane zone of an 8hPI Viral Factory at high magnification.** Dual axis STEM tomogram of a 320 nm thick section. The 3D surface rendered capsid shown in panels 7A–C was generated on the basis of the tomogram. Movie S13 was created with the slicer mode in the IMOD program, where each section is a 3.64 nm averaged view of 5 subsequent orthographic sections.
(AVI)

Movie S14 **3D surface rendering of a late-stage assembling capsid.** Dual axis STEM tomogram of a 320 nm thick section. The 3D surface rendered capsid shown in panels 7A–C was generated on the basis of the tomogram. Movie S14, demonstrating the final capsid generation stages, was created with the Aviso movie-maker platform.
(MPG)
