# Supplementary figures and images for: Membrane Assembly during the Infection Cycle of the Giant Mimivirus
Source: PLoS Pathog. 2013 May 30;9(5):e1003367. doi: 10.1371/journal.ppat.1003367 (PMC3667779; doi:10.1371/journal.ppat.1003367)

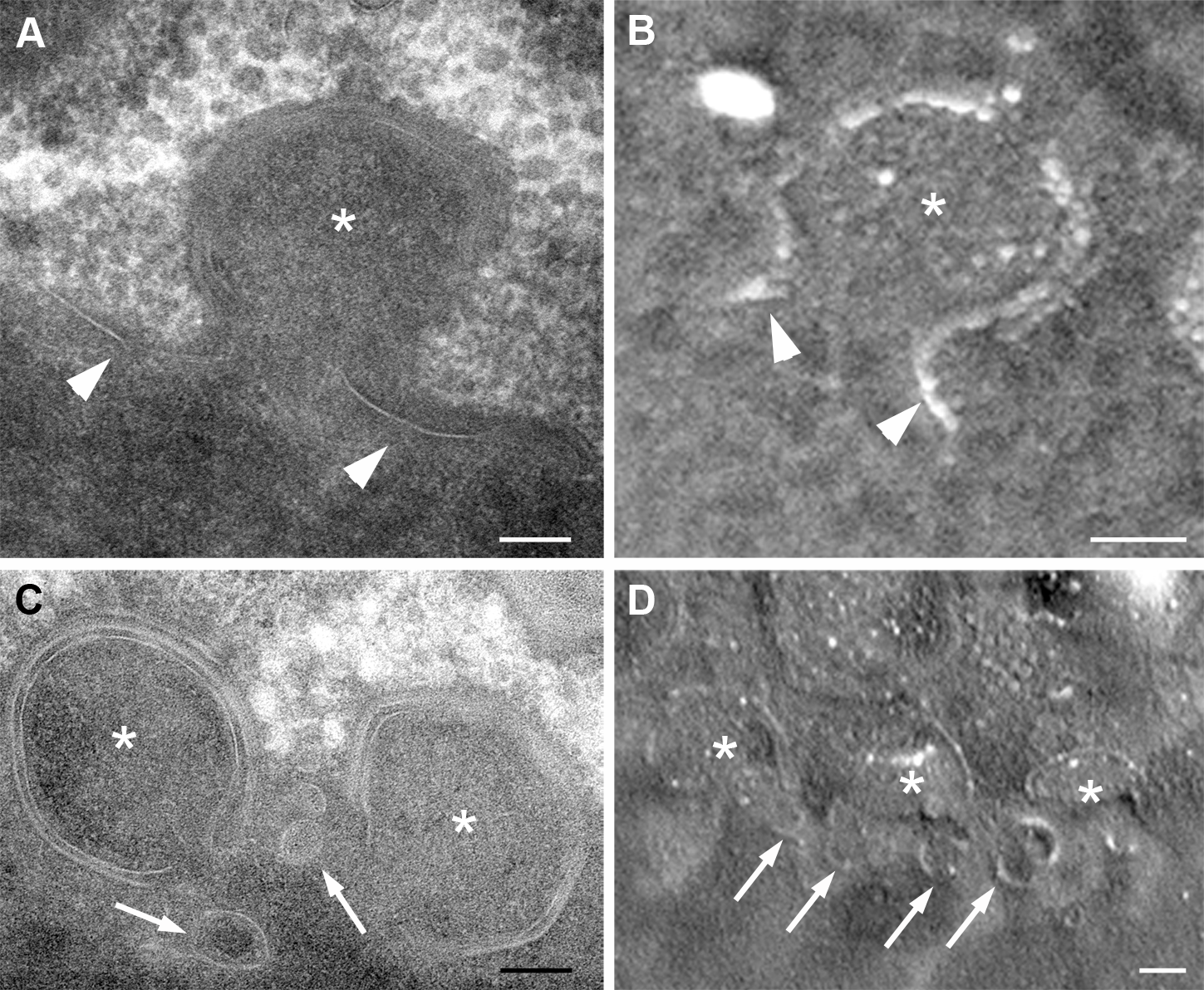

Supplement: Figure S1 — Membrane sheets and vesicles are associated with assembling capsids at 8hPI at the Viral Factory periphery. A. polyphaga cells were infected at MOI of 10 and fixed by high pressure freezing. Cells were further processed for TEM studies (A,C) or for freeze fracture cryo-SEM studies (B, D). A–D. Membrane components are found at the interface between the VF and the assembling icosahedral capsids (white asterisks) in the forms of open continuous sheets (A, B) as well as vesicles (C, D). Scale bars are 100 nm. (TIF) [file ppat.1003367.s001.tif]

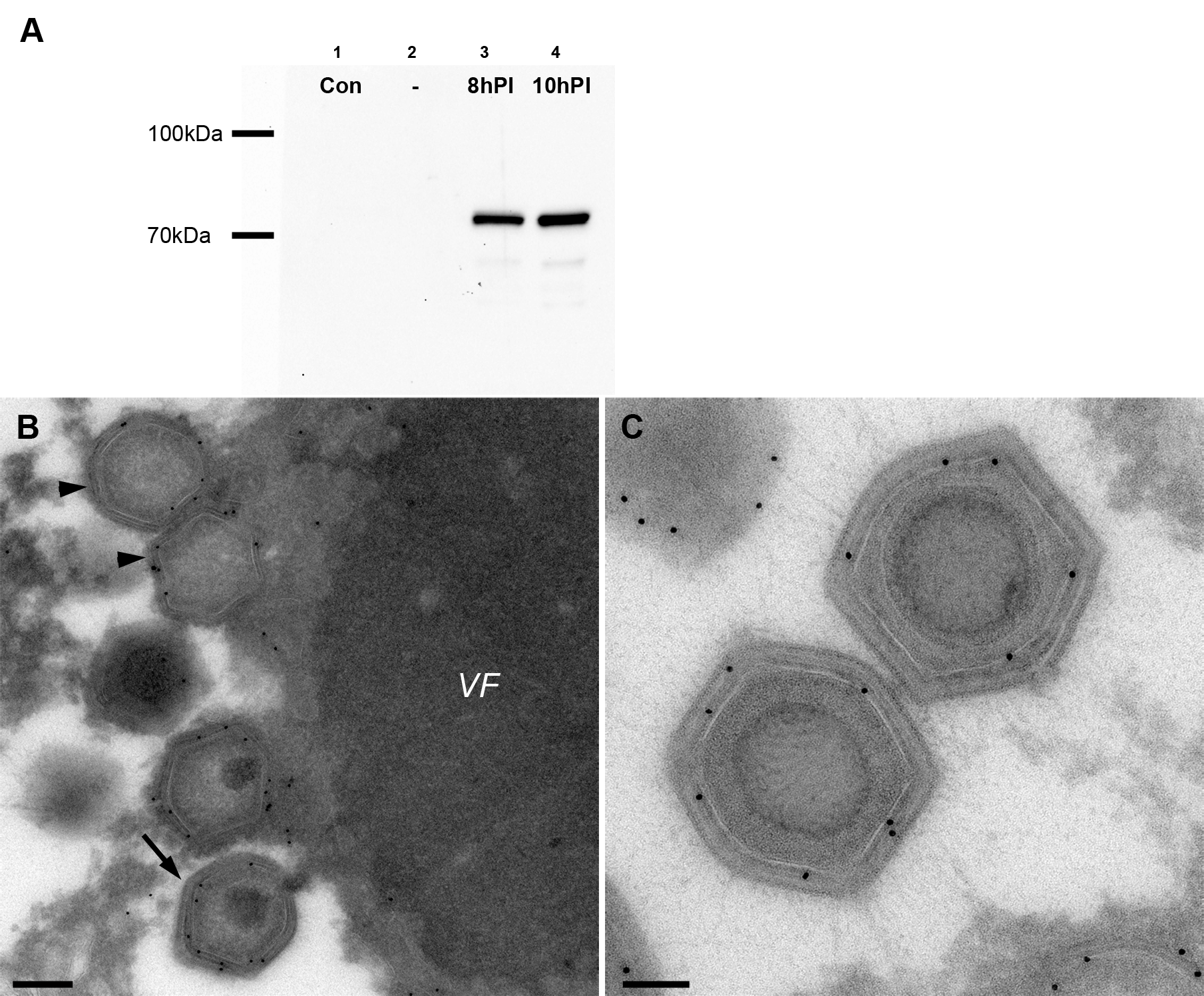

Supplement: Figure S2 — Characterization of the anti-L425 antibody by Western blot. A. A. polyphaga cells were mock infected (lane 1- Con), or infected in an MOI of 10 (lanes 3, 4). Cells were scraped from the dishes at 8hPI (lane 3) and 10hPI (lane 4) and resuspended in cold lysis buffer. Total protein concentration was measured and identical protein amounts (20 µgr per lane) were loaded on 10% SDS-PAGE. The samples were blotted onto nitrocellulose membrane. L425 protein was detected by Rabbit anti-L425, followed by HRP-conjugated antibodies and ECL reaction. Left: molecular size markers in kDa. B, C. Immuno-TEM studies of chemically fixed cryo-thawed sections of 8hPI assembling viral factory labeled with rabbit anti-L425 antibodies. Empty, fully assembled capsids (arrowheads in B) and encapsidating particles (arrow in B) are labeled with the anti L425. The L425 is also found in fully mature virions (C). Scale bars: 100 nm. (TIF) [file ppat.1003367.s002.tif]
